# Effects of *Fuzhuan* Brick-Tea Water Extract on Mice Infected with *E. coli* O157:H7

**DOI:** 10.3390/nu7075218

**Published:** 2015-07-01

**Authors:** Yuanliang Wang, Aiqing Xu, Ping Liu, Zongjun Li

**Affiliations:** 1 College of Food Science and Technology, Hunan Agriculture University, Changsha 410128, China; E-Mails: wang@hunau.net (Y.W.); xuaiqing003@163.com (A.X.); lp102700@163.com (P.L.); 2 Hunan Province Key Laboratory of Food and Biotechnology, Changsha 410128, China; 3 School of Life Science, Hunan University of Science and Technology, Xiangtan 411201, China; 4 Functional Food Sub-center, National Research Center of Engineering &Technology for Utilization of Functional Ingredients from Botanicals, Hunan Agricultural University, Changsha 410128, China

**Keywords:** *Fuzhuan* brick-tea, *E. coli* O157:H7, immunologic function, colonic microbiota, function

## Abstract

*Fuzhuan* brick-tea extract (FBTE) affects the physiology of mice infected with *Escherichia coli* O157:H7. For 10 consecutive days, 0.05, 0.5, and 1.0 g/mL FBTE was administered intragastrically to three groups of infected Kunming mice, and changes in immunological function, hematology, and histopathology were examined. The results revealed upregulation of platelets, total protein, and albumin along with downregulation of serum triglycerides, aspartate aminotransferase, creatinine, and urea nitrogen in FBTE-treated mice. Histological sections of stomach, kidney, duodenum, ileum, and colon suggested that infected mucous membranes could be rehabilitated by low- and high-dose FBTE and that inflammation was alleviated. Similarly, increased thymic function in mice treated with middle- and high-dose FBTE led to elevated serum hemolysin antibody titer and increased CD4^+^ and CD8^+^ T cells, as indicated by CD4^+^ and CD8^+^ expression on intestinal mucosa. Monocyte and macrophage function was improved by three FBTE dosages tested. Colonic microbiota analysis by denaturing gradient gel electrophoresis (DGGE) revealed characteristic bands in infected mice treated with middle- and high-dose FBTE and increased species diversity in *Lactobacillus*, *Bacteroides*, and *Clostridium* cluster IV. These results suggest that FBTE may protect kidney and liver of mice infected with *E. coli* O157:H7, improve immune function, and regulate the colonic microbiota.

## 1. Introduction

*Fuzhuan* brick-tea is a fermented tea and a high-grade variety of ancient dark tea. It is produced mainly in the Hunan, Hubei, Sichuan, Guangxi, and Yunnan provinces in China. Dark tea production has a long history, and it was transported to Western Asia and Europe via the Ancient Tea and Horse Caravan Road and the Great Silk Road. It is considered a daily necessity for the Mongolians, Uygurs, and Tibetans who reside in the frontier regions in China. It serves as an important source of vitamins, trace elements, and primary compound preparations for disease prevention [1].

**Figure 1:**
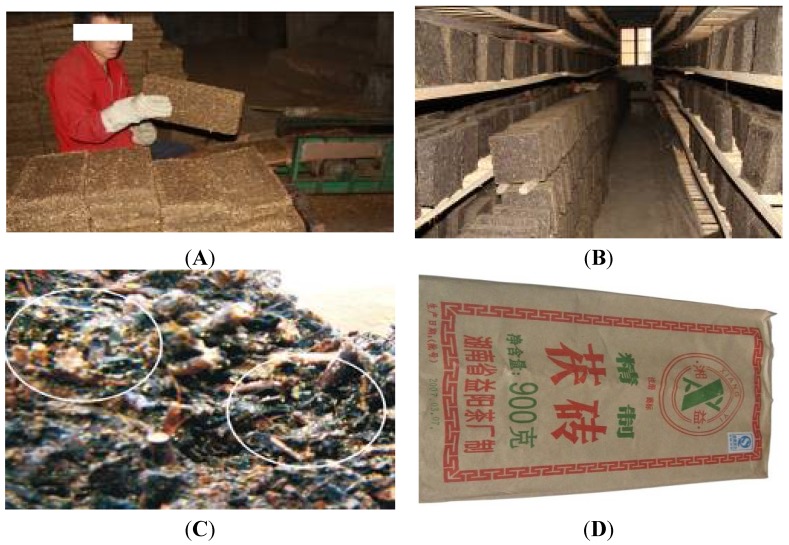
Preparation of *Fuzhuan* brick-tea. (**A**) The brick shape. (**B**) The fermentation room. (**C**) The golden flower in the fermented tea. (**D**) Packaged brick-tea.

The production process of *Fuzhuan* brick-tea is shown in Figure 1. Selected high-quality raw dark tea is steamed for 50 s at 98–102 °C and fermented for 3–4 h. Next, up to 2 kg of fermented tea is weighed, added to 250 g tea juice (water content up to 23%–26%), and pressed and molded into a brick shape (Figure 1A). Conveyor belts take the tea bricks to a fermentation room (Figure 1B), where they are kept at 26–28 °C for 22–25 days to culture *Eurotium cristatum*, also called “golden flower”, which is characteristic of *Fuzhuan* brick-tea (Figure 1C). The temperature is then increased to 38–42 °C, the water content decreases to 14%, and the tea is finally packaged in a special paper after cooling (Figure 1D).

The percentage of polyphenol extracted from ten types of dark tea samples was determined to be 24.79%–37.51%. Approximately 21–35 types of compounds were identified, including volatile oils, alcohols, aldehydes, ketones, phenols and acids, among which hexadecanoic acid and linalool compounds were relatively abundant. The flavonoid, theaflavin, caffeine, and free amino acid content from 23 varieties of dark tea samples was 0.083%, 6.445%, 7.295%, and 1.5%, respectively [2].

According to medical research scientists, biologists, and tea and food science experts over the last two decades, tea not only possesses radiation-resistant, antibacterial, antitumor [3], and antioxidant [4] effects but also plays a role in controlling cardiovascular disease [5] and in reducing body weight [6]. And those can be enhanced by the fermentation of *Eurotium cristatum.*

In addition to the health-preserving functions of the brick-tea itself, it also has unique nutritional and pharmacological functions owing to the growth of microorganisms during its unique production process; this microbial growth results in significant changes in the active substances. This study aims to investigate the effect of *Fuzhuan* brick-tea (FBTE) water extracts on mice infected with *Escherichia coli* O157:H7. These experiments provide a theoretical foundation for the pharmacological health effects of *Fuzhuan* brick-tea and promote its development and application.

## 2. Materials and Methods

### 2.1. Materials

*E. coli* O157:H7 (strain 21530) was purchased from the China Center of Industrial Culture Collection. The *Fuzhuan* brick-tea named Jingxiangyi was obtained from a tea-processing factory in Yiyang, Hunan, China. The Kunming mice (IRB number SCXX (Xiang) 2009-0004) (body weight 20 ± 2 g, 1:1 male:female) and mouse pellet feed were purchased from Silaikejingda Experimental Animal Co. Ltd. (Changsha, China). Mice were fed in a standard experimental animal house in the College of Animal Science, Hunan Agricultural University.

### 2.2. Methods

#### 2.2.1. Preparation of *Fuzhuan* Brick-Tea Water Extract

One-hundred grams of *Fuzhuan* brick-tea were ground, immersed in 400 mL distilled water, boiled for 15 min, cooled to room temperature for 1 h, filtered, and finally adjusted to a total volume of 100 mL to produce high-dose FBTE (1 g/mL). The middle (0.5 g/mL) and low (0.05 g/mL) doses of FBTE were made by diluting high-dose FBTE with sterile saline.

#### 2.2.2. Main Components Analysis

Concentration of the main components of FBTE was determined after preparation. The main components are total amino acids, polyphenols, total flavones, caffeine and soluble sugar. Total amino acids were determined with gas chromatography-mass spectrometry [7], tea polyphenols determined by ferrous tartrate and Folin-Ciocalteu methods [8], total flavones analyzed by HPLC [9], caffeine by gas chromatography [10], and soluble sugars by HPLC derivatization [11].

#### 2.2.3. Preparation of *E. coli* O157:H7 for Mouse Infection

Two mililiters of *E. coli* O157:H7 seed suspension was inoculated into 100 mL of lysogeny broth and cultivated in a shaker for 18 h at 37 °C on 120 rpm. The liquid culture was then centrifuged on 4000 *g* and washed three times with sterile saline to harvest cells. Based on the results of the pre-experiment, an appropriate volume of sterile saline was added to the cell pellet to make a cell suspension (OD_600_ = 1.0, approximately to 1.2 × 10^8^ colony forming units (CFUs) per mL) that was used to inoculate the mice.

#### 2.2.4. Animal Experiments

One-hundred and ninety mice were randomly divided into five groups after adaptive feeding for three days. The mice were placed into normal (Group A), infection model (Group B), low-dose (Group C, 1 g/kg/day), middle-dose (Group D, 10 g/kg/day) and high-dose (Group E, 20 g/kg/day) groups. Given that the recommended daily consumption of *Fuzhuan* brick-tea is no more than 8 g for adults (60 kg), the dosage in groups C, D and E is roughly equivalent to this, or 10 or 20 times higher than, respectively, the recommended amount for a human adult. FBTE was administered intragastrically together with an *E. coli* O157:H7 cell suspension (0.4 mL per mouse, or 4.8 × 10^8^ CFUs per mouse). Mouse feces were taken aseptically, and *E. coli* were detected using a test kit (Singlepath^®^ *E. coli* O157 Lateral Flow Assay, Merck KGaA, Darmstadt, Germany). The results confirmed that groups B–E had been successfully infected by the pathogenic bacterium. Thereafter, groups C, D, and E were treated with low-, middle-, and high-dose FBTE intragastrically (0.4 mL per mouse) once daily at the same time each day. Group B was administered sterile saline (0.4 mL per mouse). The mice had free access to water and food during the experimental period and were sacrificed after 10 days.

#### 2.2.5. Index of Immune Organs

Mice were sacrificed by cervical vertebra dislocation, and the spleen and thymus were removed. The blood on organ surfaces was drained with filter paper before weighing. The immune organs index (IOX) was calculated using the following formula:

IOX = weight of immune organs (mg)/body weight (g)(1)

#### 2.2.6. Test of Monocyte/Macrophage Phagocytic Function

The caudal vein was injected with India ink (0.1 mL/10 g body weight). After 2 and 10 min, 20 μL of blood were taken from the plexus venae angularis and added to 2 mL of 0.1% Na_2_CO_3_ solution. The optical density (OD) at 600 nm was taken by spectrophotometer. The carbon clearance index (*K*) and the phagocytosis coefficient (a) were calculated as follows:

*K* = (logOD_2_ − logOD_10_)/(*t*_10_ − *t*_2_) (2)

a = (body weight/spleen weight plus liver weight) × (*K*)^1/3^ (3)

#### 2.2.7. Blood Analysis

Hemoglobin, red and white blood cells, and platelets were detected with an animal blood cell analyzer. Blood biochemical parameters such as total protein (TP), albumin (ALB), alanine aminotransferase (ALT), aspartate aminotransferase (AST), creatinine (CREA), blood urea nitrogen (UREA), total cholesterol (AST), triglyceride (TG), and glucose (Glu) were measured with an automatic biochemical analyzer.

#### 2.2.8. Determination of Serum Hemolysin

Previously prepared sheep blood was washed with physiological saline and centrifuged three times for 10 min at 2000 rpm to obtain sheep red blood cells (SRBC). Physiological saline was added to make a 2% SRBC suspension. Ten mice from each group were randomly selected and immunized with 2 mL of SRBC by intraperitoneal injection. Blood was collected four days later by orbital bleeding into a centrifuge tube. Different dilutions of blood serum were prepared by centrifugation and dilution with physiological saline. Hemagglutination was determined by blending 100 μL of serum (various dilutions) with 100 μL 0.5% SRBC suspension in micro-hemagglutination test plates and incubating at 37 °C for 3 h.

#### 2.2.9. Determination of CD4^+^ T and CD8^+^ T Cell Numbers in Intestinal Mucosa by Immunohistochemistry

Samples of duodenum, ileum, and colon from 10 mice in each group were collected, fixed in 10% formalin solution, dehydrated, embedded in paraffin, sectioned, and dewaxed in water. Sample sections were placed in 0.3% H_2_O_2_ for 15 min to block endogenous peroxidase. The primary antibody (RabMAb^®^—Rabbit monoclonal antibody, Abcam, Cambridge, MA, USA) was added, and the samples were incubated overnight in a moist box at 4 °C. The secondary antibody (RabMAb^®^—Rabbit monoclonal antibody HRP, Abcam) was then added, and the samples were incubated for 1 h at room temperature. DAB (3,3′-diaminobenzidine) colorimetric reactions were generated, stained with hematoxylin, and sealed in neutral gum. Controls were treated identically except that PBS was substituted for the primary antibody. Four sections of duodenum, ileum, and colon from each mouse were used to determine the number of CD4^+^ T and CD8^+^ T cells in the intestinal mucosa using a light microscope.

#### 2.2.10. Genomic DNA Extraction from Mice Feces

The mice feces were collected in sterile bags and kept on ice, immediately taken to the laboratory and subsequently frozen at −20 °C.

The frozen samples (0.25 g) were transferred to a sterile tube (50 mL) adding 5 mL of ice-cold 0.05 M phosphate-buffered saline (PBS) (150 mM NaCl, 10 mM Na_2_HPO_4_, 20 mM NaH_2_PO_4_, pH 7.4), and homogenized by the addition of 5 sterile glass beads (3 mm in diameter), vortexed fully for 3 min on 8000 rpm. Samples were centrifuged at 400× *g* for 2 min, and the supernatants were transferred to new 50-mL sterile tubes. Pellets were resuspended in 3 mL PBS buffer, mixed thoroughly and centrifuged at 400× *g* for 2 min. All the suspensions were transferred to new 50-mL sterile tubes and mixed with 3 voL of 4% polyformaldehyde, incubated on ice for 1 h. Following fixation, the cell suspension was centrifuged at 8000× *g* for 3 min and the cell pellet was resuspended in 4 mL PBS buffer, mixed with 4 mL absolute ethanol and stored at −20 °C for 20 min. Subsequently, the cell pellet was harvested by centrifugation at 8000× *g* for 3 min and resuspended in 4 mL of TE buffer (pH 8.0) for use. The detailed extraction protocol used was the same as that described as method two by Tang *et al.* [12].

#### 2.2.11. Polymerase Chain Reaction (PCR) Amplification of the 16S rDNA Fragment for DGGE Analysis of Mouse Colonic Microbiota

Four primer pairs were used for denaturing gradient gel electrophoresis (DGGE) analysis to detect changes in mouse colonic bacterial diversity. The predominant bacteria were *Lactobacillus*, *Bifidobacterium*, *Bacteroides*, and *Clostridium* cluster IV. Approximately 500–700 nucleotides of the different 16S rDNA target regions were amplified. All primer sequences are listed in Table 1. Primers were synthesized by Sangon Biotech Co. Ltd. (Shanghai, China). PCR was performed using a MyCycler thermal cycler (Bio-Rad, Hercules, CA, USA), and amplifications were conducted in a final volume of 50 μL containing 5.0 μL of 10× PCR buffer (Mg^2+^-free), 5.0 μL of 25 mM MgCl_2_, 1 μL of dNTP mixture (10 mM each dNTP), 0.5 μL of Taq polymerase (5 units/μL, Promega, Shanghai, China), 1.0 μM of each primer, 1 μL of extracted DNA [13] and sterilized distilled water. Reactions were run for 30 cycles of denaturation at 95 °C for 60 s, annealing at 55 °C for 45 s, and extension at 72 °C for 60 s. Initial denaturation steps at 95 °C for 5 min and final extension steps at 72 °C for 10 min were also conducted. A 5-μL sample of each PCR product was analyzed by 1% agarose gel electrophoresis in 0.5× Tris-borate-EDTA (TBE) buffer.

**Table 1:** Primers for PCR amplification of different bacterial groups.

Specificity	Primer	Primer Sequence	Ref.
Total bacteria	F-968-GC	GC-clamps-5′-AACGCGAAGAACCTTAC-3′	[14]
	R-1401	5′-CGGTGTGTACAAGACCC-3′	
*Lactobacillus*	Lac2GC	GC-clamps-5′-AACGCGAAGAACCTTAC-3′	[15]
	Lac1	5′-CGGTGTGTACAAGACCC-3′	
*Bacteroides*	32f-GC	GC-clamps-5′-AACGCTAGCTACAGG CTT-3′	[16]
	708r	5′-CAATCGGAGTTCTTCG-3′	
*Clostridium* cluster IV	sg-Clep-F-GC	GC-clamps-5′-GCACAAGCAGTGGAGT-3′	[17]
	sg-Clep-R	5′-CTTCCTCCGTTTTGTCAA-3′	

The DCode Universal Mutation Detection System (Bio-Rad, Hercules, CA, USA) was used for DGGE analysis. Each PCR product was mixed with an equal volume of 2× loading buffer and loaded into the sample wells. Electrophoresis was performed in polyacrylamide gels (8% (w/v) acrylamide-bisacrylamide at 37.5:1) with a 30%–70% denaturing gradient (100% corresponding to 7 M urea and 40% (w/v) deionized formamide) that increased in the direction of the electrophoretic run [18]. The DGGE fingerprint map was generated by unweighted pair group method with arithmetic mean (UPGMA) analysis using the Quantity One 1-D Analysis software (Bio-Rad).

#### 2.2.12. Statistical Treatment

The data are presented as the mean ± SD. Statistical evaluation was performed using SPSS 11.5 software (IBM, Chicago, IL, USA) to conduct independent *t*-tests between sample pairs among treatment, normal, and model groups. Means were considered significantly different at *p* < 0.05.

### 2.3. Ethical Statement

All procedures were followed by the guidelines of the Hunan Province Key Laboratory of Food and Biotechnology at Hunan Agriculture University, and the ethical approval number is 201001001 (approved on 5 January 2010).

## 3. Results

### 3.1. Concentrations of Main Components of FBTE

The high-dose FBTE was used as the sample for components analysis. All determination results showed that soluble sugar is the main component in FBTE, followed by tea polyphenols, total flavones, and caffeine; total free amino acids are at the lowest level of the components. The precise content is given in Table 2.

**Table 2:** Concentrations of main components of *Fuzhuan* brick-tea extract (g/100 mL).

Total Free Amino Acids	Tea Polyphenols	Total Flavones	Caffeine	Soluble Sugar
0.25	3.89	2.43	1.12	4.20

### 3.2. Clinical Manifestation in Mice

The results showed that control mice (Group A) were active, had normal fur color, and forcefully resisted tail grasping during the experiment. However, *E. coli*-infected mice (Groups B–E) demonstrated reduced activity, lack of appetite, fluffy fur, and weak resistance to tail grasping. In mice receiving FBTE (Groups C–E), the above symptoms were gradually relieved three days after infection, and most mice essentially recovered.

### 3.3. Blood Cell Tests

Blood cell results for each of the five groups are shown in Table 3. The number of white blood cells (WBC) in the infection model and FBTE groups increased. The low-dose group had higher WBC counts than the control group (*p* < 0.05). Although the number of platelets decreased in the model and all FBTE-treated groups, platelet counts in the infection model and low-dose groups were significantly lower than that of controls (*p* < 0.05).

**Table 3:** Results of blood cell tests of mice from each of the five groups (*n* = 10, $\bar{x}$ ± s).

Groups	White Blood Cells (WBC) (×10^9^/L)	Red Blood Cells (RBC) (×10^12^/L)	Hemoglobin (HGB) (g/L)	Platelets (PLT) (×10^9^/L)
Normal (A)	0.89 ± 0.24	8.42 ± 3.79	125.25 ± 14.80	1295.70 ± 264.84
Model (B)	1.44 ± 0.81	6.04 ± 3.86	120.15 ± 21.55	652.05 ± 240.31 ^∆^
Low dose (C)	2.08 ± 0.99 ^∆^	7.09 ± 4.45	119.70 ± 14.29	747.15 ± 139.72 ^∆^
Middle dose (D)	1.42 ± 0.54	6.12 ± 4.5	123.60 ± 22.80	872.10 ± 193.55
High dose (E)	1.46 ± 0.92	9.07 ± 3.26	122.40 ± 23.33	1019.28 ± 142.57

^∆^ *p* < 0.05 *vs.* normal group.

### 3.4. Blood Biochemistry

The middle- and high-FTBE dosages both improved total protein content in infected mice, but the middle dose was more effective (*p* < 0.05). The middle dose improved albumin content, with a significant difference between groups B and D (*p* < 0.01). All the groups receiving FBTE exhibited decreased serum triglycerides (*p* < 0.05) compared to both the normal and infection model groups. None of the five groups exhibited significant differences in TC (total cholesterol), BS (blood sugar), or ALT (alanine aminotransferase). AST (aspartate transaminase), however, was significantly higher in the infection model group than in controls (*p* < 0.01), and it was significantly reduced in the middle- and high-dose FBTE groups (*p* < 0.05); data shown in Table 4.

**Table 4:** Results of blood biochemical tests of mice from each of the five groups (*n* = 10, $\bar{x}$ ± s).

Groups	Total Protein (TP) (g/L)	Albumin (ALB) (g/L)	Triacylglycerol (TG) (mmoL/L)	Total Cholesterol (TC) (mmoL/L)	Blood Sugar (BS) (mmoL/L)	Alanine Aminotransferase (ALT) (U/L)	Aspartate Transaminase (AST) (U/L)	Blood Urea Nitrogen (UREA) (mmoL/L)	Creatinine (CREA) (µmoL/L)
Normal (A)	54.98 ± 7.34	41.72 ± 5.28	1.55 ± 0.27	3.08 ± 0.93	3.28 ± 1.18	68.80 ± 16.63	142.21 ± 27.46	5.92 ± 1.62	46.20 ± 5.14
Model (B)	51.58 ± 5.82	38.29 ± 4.07	1.30 ± 0.33	2.90 ± 0.71	3.59 ± 1.53	82.53 ± 15.29	205.64 ± 63.45 ^★^	8.80 ± 2.62 ^∆^	49.24 ± 4.63
Low dose (C)	51.55 ± 8.36	38.27 ± 6.13	0.99 ± 0.26 ^★^^,▲^	3.00 ± 0.89	3.04 ± 1.04	68.93 ± 19.89	178.43 ± 41.73	8.33 ± 4.51 ^∆^	46.35 ± 8.07
Middle dose (D)	61.08 ± 7.59 ^▲^	46.26 ± 3.41 *	0.96 ± 0.37 ^★^^,▲^	2.63 ± 0.99	3.45 ± 1.74	63.33 ± 11.19	160.44 ± 31.30 ^▲^	6.87 ± 2.04	42.30 ± 10.10 ^▲^
High dose (E)	56.50 ± 13.80	43.09 ± 7.65	0.87 ± 0.14 ^★^^,▲^	2.42 ± 0.50	3.64 ± 1.70	55.20 ± 12.76	167.58 ± 32.21 ^▲^	5.49 ± 0.65 *	42.91 ± 3.88 ^▲^

^∆^ *p* < 0.05 *vs.* normal group; ^★^ *p* < 0.01 *vs.* normal group; ^▲^ *p* < 0.05 *vs.* model group; * *p* < 0.01 *vs.* model group.

UREA content rose in the infection model group compared with controls (*p* < 0.05), and all mice administered FBTE displayed a gradual decrease in UREA content. UREA content was particularly decreased in mice treated with high-dose FBTE compared to the infection model mice (*p* < 0.01). CREA content did not vary significantly in the infection model mice relative to controls. However, CREA in the middle- and high-dose groups significantly decreased relative to the infection model group (*p* < 0.05).

### 3.5. Effects of FTBE on Immune Organ Indices

The thymus index of the middle and high-dose groups was higher than that of the infection model group (*p* < 0.05), and the middle-dose group was quite different from with the infection model group (*p* < 0.01) (Table 5). Significant differences in the spleen index among the groups were not observed.

**Table 5:** Effects of *Fuzhuan* brick-tea extract on thymus and spleen indices ($\bar{x}$ ± s).

Groups	Samples	Thymus Index	Spleen Index
Normal (A)	10	2.75 ± 0.39	3.67 ± 0.97
Model (B)	10	2.47 ± 0.87	3.69 ± 0.91
Low dose (C)	10	2.99 ± 0.89	4.02 ± 0.75
Middle dose (D)	10	3.38 ± 0.76 *	3.96 ± 0.85
High dose (E)	10	3.16 ± 0.64 ^▲^	3.80 ± 0.79

^▲^ *p* < 0.05 *vs.* model group; * *p* < 0.01 *vs.* model group.

### 3.6. Changes in Mononuclear Macrophage Function

A significant difference in mononuclear macrophage function was observed between the infection model and normal groups (*p* < 0.01), and all FBTE-treated groups were significantly different from the infection model group (*p* < 0.05), particularly the high- and middle-dose FBTE groups (*p* < 0.01) (Table 6).

**Table 6:** Effect of *Fuzhuan* brick-tea extract on mononuclear macrophage immune function ($\bar{x}$ ± s).

Groups	Samples	Phagocytosis Coefficient
Normal (A)	10	5.07 ± 0.76
Model (B)	10	3.93 ± 0.50 ^★^
Low dose (C)	10	4.92 ± 0.99 ^▲^
Middle dose (D)	10	5.31 ± 1.14 *
High dose (E)	10	5.27 ± 0.73 *

^★^ *p* < 0.01 *vs.* normal group; ^▲^ *p* < 0.05 *vs.* model group; * *p* < 0.01 *vs.* model group.

### 3.7. Changes in Serum Hemolysin

A difference in hemolysin between the infection model and normal groups was observed (*p* < 0.05). The serum anti-SRBC antibody level in the middle- and high-dose FBTE groups was elevated (*p* < 0.05), as was the low-dose group (*p* < 0.01) (Table 7).

**Table 7:** Effect of *Fuzhuan* brick-tea extract on serum hemolysin in mice ($\bar{x}$ ± s).

Groups	Samples	Antibody Volume
Normal (A)	10	44.60 ± 5.80
Model (B)	10	37.20 ± 6.30 ^∆^
Low dose (C)	10	42.50 ± 9.35
Middle dose (D)	10	45.50 ± 8.58 ^▲^
High dose (E)	10	46.70 ± 8.41 *

^∆^ *p* < 0.05 *vs.* normal group; ^▲^ *p* < 0.05 *vs.* model group; * *p* < 0.01 *vs.* model group.

### 3.8. Effect of FBTE on CD4^+^ and CD8^+^ Lymphocyte Number in Intestinal Tract Mucous Membranes

Immunohistochemical staining was used to detect CD4^+^ and CD8^+^ T cells in the duodenum, ileum, and colon. CD4^+^ T lymphocytes were located in the lamina propria of the intestinal mucosa, with a few also present in the epidermis. CD8^+^ T-lymphocytes were located in both the stratum basale and lamina propria of the intestinal mucosa. Cell counting showed that the number of CD4^+^ and CD8^+^ T cells in the infection model group were lower than in the normal group (*p* < 0.05), and the middle- and high-dose FBTE groups exhibited enhanced numbers (*p* < 0.05) compared with the infection model group (Table 8).

**Table 8:** Effect of FBTE on the number of CD4^+^ and CD8^+^ T lymphocytes ($\bar{x}$ ± s).

Groups	Samples	CD4^+^ T	CD8^+^ T
Normal (A)	10	203.50 ± 28.32	144.80 ± 23.93
Model (B)	10	167.00 ± 35.00 ^∆^	117.10 ± 22.69 ^∆^
Low dose (C)	10	186.30 ± 34.70	133.30 ± 24.49
Middle dose (D)	10	211.90 ± 50.49 ^▲^	150.20 ± 39.54 ^▲^
High dose (E)	10	206.60 ± 40.48 ^▲^	145.90 ± 31.67 ^▲^

^∆^ *p* < 0.05 *vs.* normal group; ^▲^ *p* < 0.05 *vs.* model group.

### 3.9. DGGE Analysis of Mouse Colonic Microbiota

**Figure 2:**
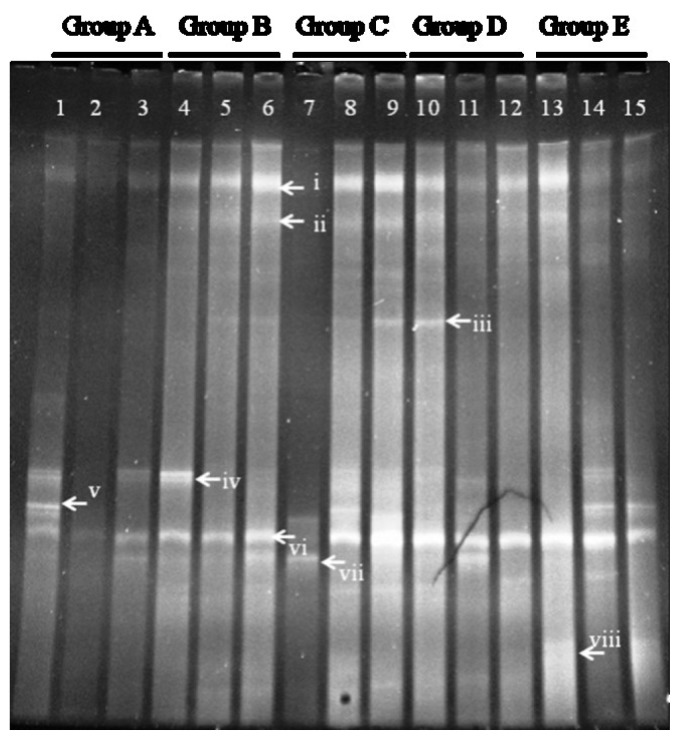
DGGE fingerprint for the total bacterial microbiota obtained from mouse distal colon. Lanes 1–3 (Group A) represent normal controls. Lanes 4–6 (Group B) represent infection models. Lanes 7–9 (Group C) represent infected mice treated with low-dose FTBE. Lanes 10–12 (Group D) represent infected mice treated with middle-dose FBTE, and lanes 13–15 (Group E) represent infected mice treated with high-dose FBTE. The arrows (i–viii) indicate bands that changed in intensity.

**Figure 3:**
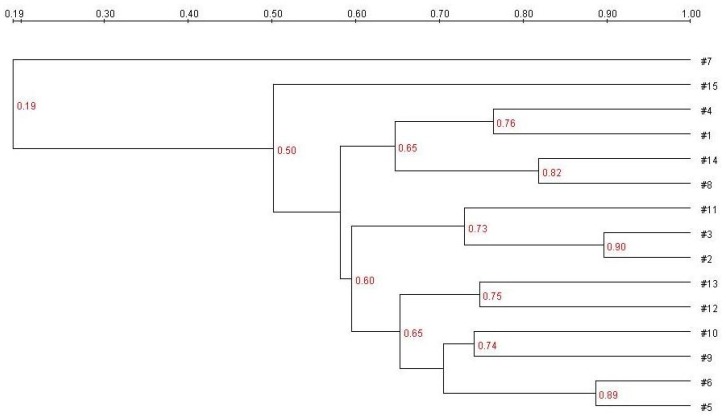
Cluster analysis using the unweighted pair group method with arithmetic mean (UPGMA) method based on the Dice coefficient for the band pattern in Figure 2.

DGGE analysis of the total colonic microbiota was targeted on the V6–V8 region of 16S rDNA. The primary bacterial fingerprints are shown in Figure 2. Results from all four treatment groups were different from controls. The *E. coli* treatment (Group B) generated more bands in the DGGE fingerprint than seen in Group A. Some bacteria (denoted by bands i, ii, iii, vi, and vii) increased in quantity, and some (band v) decreased. The band pattern also changed depending on the FBTE concentration. In the low-dose group (Group C), band v appeared similar to the control, whereas in the middle-dose group (Group D), band iii varied among different mice; band iii was not present following high-dose FBTE treatment (Group E). Band v reappeared in some Group E mice (similar to Group A), and a new band (viii) was observed. These results suggest that new bacteria were being propagated in the colon. However, according to UPGMA analysis, the 15 lanes did not correspond to the five groups (Figure 3). The DGGE map suggests the existence of individual differences in the colonic microbiota of the 15 mice. We conclude, therefore, that high-dose FBTE is beneficial to infected mice and that the bacteria represented by band iii are eliminated.

**Figure 4:**
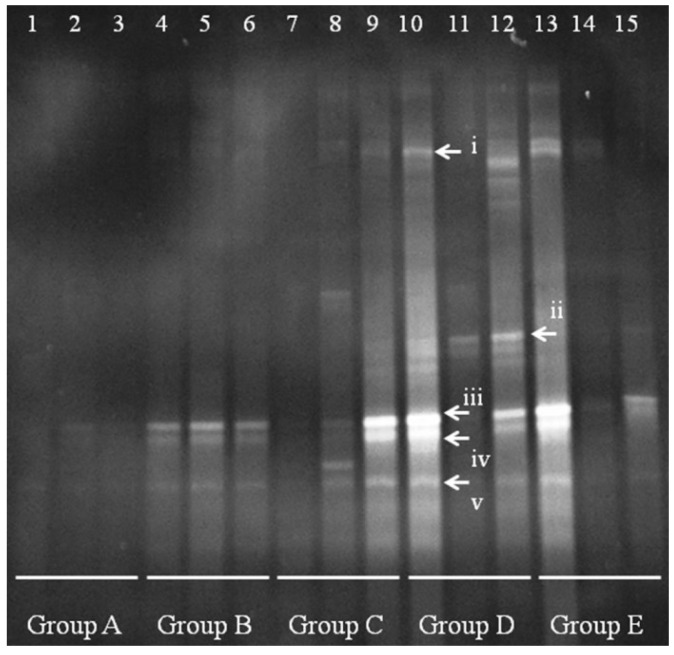
Denaturing gradient gel electrophoresis (DGGE) for *Lactobacillus* obtained from mouse distal colonic microbiota. Lanes 1–3 (Group A) represent normal controls. Lanes 4–6 (Group B) represent infection models. Lanes 7–9 (Group C) represent infected mice treated with low-dose FTBE. Lanes 10–12 (Group D) represent infected mice treated with middle-dose FBTE, and lanes 13–15 (Group E) represent infected mice treated with high-dose FBTE. The arrows (i–v) indicate bands that changed in intensity.

A clear change in the *Lactobacillus* population is apparent between Group A and Groups B–E (Figure 4). In controls (group A), the quantity and species diversity of *Lactobacillus* were present at low levels, with almost no clearly visible band. After *E. coli* infection, *Lactobacillus* fingerprints appeared in bands iii–v. In low-dose-treated mice (Group B), the band intensity increased in mice 8 and 9, but not in mouse 7 (no difference from control). This result suggests that the new *Lactobacillus* were fixed in the colon. Group D mice had some bands similar to i and ii, and the *Lactobacillus* bands iii-v increased in quantity. However, high-dose treatment (Group E) appeared to cause changes in the *Lactobacillus* bands, as seen in mice 14 and 15, with a single change in mouse 13.

**Figure 5:**
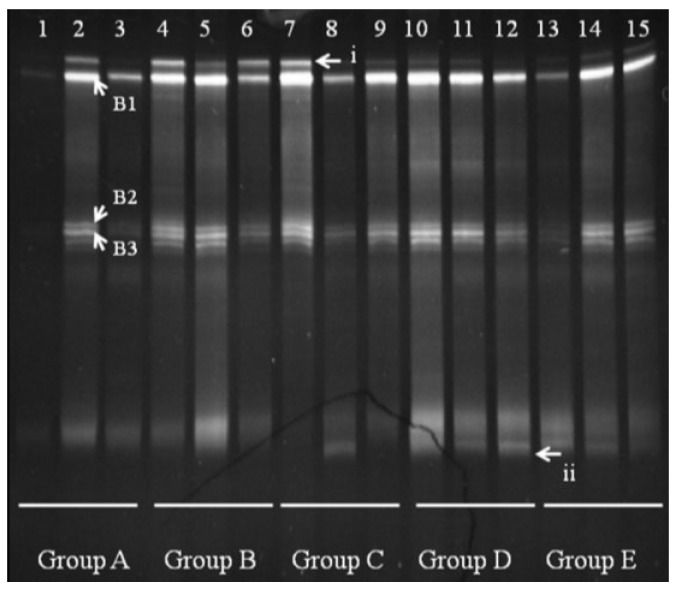
Denaturing gradient gel electrophoresis (DGGE) for *Bacteroides* obtained from mouse distal colonic microbiota. Lanes 1–3 (Group A) represent normal controls. Lanes 4–6 (Group B) represent infection models. Lanes 7–9 (Group C) represent infected mice treated with low-dose FTBE. Lanes 10–12 (Group D) represent infected mice treated with middle-dose FBTE, and lanes 13–15 (Group E) represent infected mice treated with high-dose FBTE. The arrows (i–ii) indicate bands that changed in intensity. Representative bands (B1–B3) were excised for cloning and sequencing.

We observed several *Bacteroides* bands in groups A and B, with some individual bands unique to Group A. Bands B1, B2, and B3 appeared in all groups shown (Figure 5). Sequencing and BLAST analysis of bands B1, B2, and B3 against GenBank revealed the closest relatives as *Bacteroidesuniformis*, *Bacteroidessartorii*, and *Bacteroidesacidifaciens*, respectively, which are 97% similar. Band i was reduced by FBTE treatment even at the low dose, although it appeared in all the mice in the infection model group. A new band (ii) appeared in the middle- and high-dose FBTE groups, suggesting that middle- or high-dose FBTE treatment may increase the diversity of *Bacteroides* in the colon.

**Figure 6:**
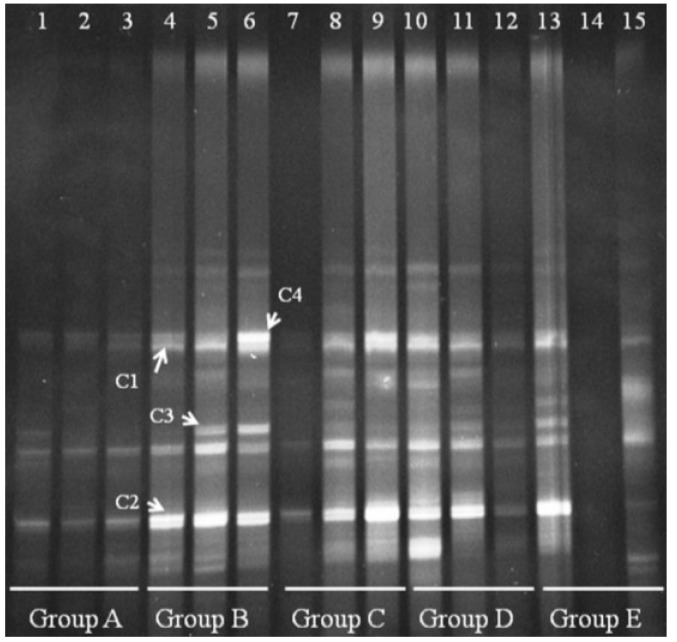
Denaturing gradient gel electrophoresis (DGGE) for *Clostridium* cluster IV obtained from the mouse distal colonic microbiota. Lanes 1–3 (Group A) represent normal controls. Lanes 4–6 (Group B) represent infection models. Lanes 7–9 (Group C) represent infected mice treated with low-dose FTBE. Lanes 10–12 (Group D) represent infected mice treated with middle-dose FBTE, and lanes 13–15 (Group E) represent infected mice treated with high-dose FBTE. Representative bands (indicated by C1–C4) were excised for cloning and sequencing.

We also performed DGGE analysis for *Clostridium* cluster IV in the distal colonic microbiota. Clear changes appeared for *Clostridium* cluster IV in Groups B–E (Figure 6). Group A bands were faint, suggesting that *Clostridium* cluster IV is present at low levels in normal mice. Four bands appeared in Group B, and we verified that the four bands were derived from *Clostridium orbiscindens* and *Clostridium viride*. All four bands could be suppressed in a few mice by treating with FBTE, but most bands were not changed. This result suggests that tea extract cannot inhibit the growth of *Clostridium* cluster IV in the colons of mice infected with *E. coli* O157:H7.

## 4. Discussion

### 4.1. Effects of FTBE on Blood Cells

The *E. coli* O157:H7 strain produces Shiga toxin. Interaction of the toxin B-subunit with the Gb3 acceptor on vascular endothelial cells inhibits eukaryotic protein synthesis, leading to endothelial cell damage and cell death.

In addition, there are lipopolysaccharides in the *E. coli* outer membrane, and certain cytokines (e.g., interleukin I and tumor necrosis factor) are produced by mouse monocytes. These components contribute to cytotoxic aggravation and endothelial cell damage, which promote blood coagulation. These pathological changes lead to elevated peripheral WBC counts. Some mice also experience a reduction in hemoglobin and platelets [19].

In this study, we observed elevated WBC counts in the infection model and in FBTE-treated groups (Table 3), indicating that an inflammatory response had occurred after infection. Blood platelets decreased from the control values in both the infection model and in FBTE-treated groups. Interestingly, the infection model and low-dose groups decreased noticeably, while platelets were restored to normal levels in the middle- and high-dose FBTE groups (Table 3). This result shows that mice were susceptible to Shiga toxin after *E. coli* infection, with clear pathophysiological changes in tissues and organs and increased platelet consumption. However, middle- and high-dose FBTE treatments effectively elevate the platelet counts and restore them to normal levels, with a significant dose-dependent relationship. Statistical analysis indicated that the number of red blood cells did not differ between groups.

### 4.2. Effects of FBTE on Blood Chemistry

Plasma protein synthesis decreases as liver cells are damaged. Therefore, determining the total protein and albumin levels in serum can reflect the liver protein metabolic function. Furthermore, when liver cells are damaged, the increase in hepatocyte membrane permeability will lead to the release of intracytoplasmic ALT and AST into plasma, which causes an increase in serum ALT and AST enzyme activity (Table 4).

The total serum protein and albumin in the infection model group was reduced relative to controls, whereas it increased for the middle-dose FBTE group. In contrast, infection model serum AST markedly increased compared with controls, but dropped in the middle- and high-dose groups (Table 4). These observations suggest that hepatocytes underwent various degrees of damage after the mice were infected with *E. coli*, resulting in altered liver function and protein synthesis. Moreover, owing to changed liver cell membrane permeability, serum AST rose. Liver function was restored after the administration of a proper FTBE dose.

Detection of serum creatinine and blood urea nitrogen is a key method for assessing kidney function [20]. The kidney tissue endured significant damage following infection because the Shiga toxin secreted by *E .coli* O157:H7 is specific for the Gb3 receptor, which is expressed at high levels in the kidney. Others have shown that Shiga toxin is cytotoxic to human kidney endothelial cells *in vitro* [21].

Following intragastric administration of *E. coli* O157:H7, CREA and UREA content rose compared with controls, whereas they declined in the middle- and high-dose FBTE groups. This demonstrated that because glomerular filtration was affected by *E. coli* O157:H7, serum creatinine and blood urea nitrogen could not be regularly discharged from the body through the urinary system, which increased their blood concentration. The decline in serum creatinine and blood urea nitrogen in the middle- and high-dose groups indicates that FBTE can restore impaired kidney function in the infection model group.

### 4.3. Effects of FBTE on Immune Organs

Thymus and spleen indices reflect the functional status of innate immunity [22]. After the mice were fed intragastrically, the thymus and spleen indices increased 5.0% and 5.2% over controls [23]. In this study, high- and middle-dose FBTE increased the thymus index but not the spleen index (Table 5). This suggests that FBTE directly stimulates thymus development but has no obvious influence on spleen.

### 4.4. Effects of FBTE on Monocyte/Macrophage Phagocytic Function

The carbon clearance test is a classic method to evaluate monocyte/macrophage phagocytic function. A certain amount of particulate carbon is intravenously injected into the mouse, and it is quickly phagocytosed by monocytes and macrophages and eliminated from the blood. Therefore, the rate of elimination from blood reflects monocyte/macrophage phagocytosis [24]. We found that the infection model group phagocytosis coefficients were lower than that of controls, and that all of the FBTE-treated groups had higher coefficients than the infection model group. More specifically, the middle- and high-dose groups were enhanced (Table 6). This suggests that the decline in monocyte/macrophage phagocytosis, which is due to the *E. coli* O157:H7 infection, can be reversed by repeated administration of FBTE.

### 4.5. Effects of FBTE on Humoral Immune Function

humoral immune function. Mice immunized with SRBC produce anti-SRBC antibodies, known as hemolysin, which dissolve red blood cells. The degree of agglutination is used to test hemolysin levels [25]. The increased serum hemolysin in the middle- and high-dose groups (Table 7) indicates that middle- and high-dose FBTE enhance mouse humoral immunity.

### 4.6. Effect of FBTE on CD4^+^ and CD8^+^ Lymphocyte Number in Intestinal Tract mucous Membranes

In this experiment, even the mice in model (B) had a healthy index in terms of the ratio of CD4^+^ and CD8^+^. All the test groups had the similar ratio of 1.4. These results indicate that the immune systems in all four groups of mice have not been changed fundamentally, but only changes in cell quantity of CD4^+^ and CD8^+^. Furthermore, the low dose does not have the function to compensate the reduction of CD4^+^ and CD8^+^ caused by *E. coli* O157:H7, but rather only has a slight positive effect; however, the middle and high dose can completely compensate for this reduction. These data (Table 8) indicate that the extract is beneficial for the proliferation of CD4^+^ and CD8^+^, even if they had been decreased by *E. coli* O157:H7.

### 4.7. Effects of FBTE on Colonic Microbiota

The DGGE results revealed individual differences in colonic microbiota. The colonic microbiota changed after *E. coli* O157:H7 infection and also with different doses of FBTE, and new bacteria even appeared to grow in the colon. It is certain that these new bacteria already existed in the normal and infection model groups but in undetectable amounts. However, after administration of tea extract, they propagated and remained in the colon. It may be that tea extract inhibits *E. coli* growth in the colon. This would be consistent with previous reports [26,27,28], indicating that tea polyphenols or catechin had bacteriocin activity and could repress the growth of some bacteria.

## 5. Conclusions

Examination of mouse peripheral blood cells in each group revealed a decreased number of platelets in mice infected with *E. coli* O157:H7. However, administering middle and high doses of FBTE to infected mice enhanced platelet number and even restored normal platelet counts in a dose-dependent manner. Blood biochemical indices indicated that administration of middle- and high-dose FBTE decreased serum triglycerides, AST, CREA, and UREA but cannot compensate blood platelets to normal level, and that middle-dose FBTE increased total serum protein and albumin.

These studies were designed to investigate the effects of FBTE on innate immunity, humoral immunity, and the local intestinal immunity of mice infected with *E. coli* O157:H7. The results show that administration of middle- and high-dose FBTE enhances the thymus index of mice, serum hemolysin levels, and the expression of CD4^+^ and CD8^+^ T cells in the intestinal mucosa. In addition, the three test dosages of tea extract improved monocyte/macrophage phagocytic function.

According to the Technical Standards for Testing and Assessment of Health Food in China (2003), when evaluating cellular immune function (including humoral immunity and the function of monocytes/macrophages and natural killer cells), if two or more functional tests are positive, the substances can be said to have immunomodulatory effects. In combination with experimental results, we demonstrate that FBTE improves mouse immunological function and that the effects are realized through multiple means and multiple targets. FBTE treatment can therefore be considered to relieve immune impairment induced by *E. coli* O157:H7 and to promote enhanced diversity of *Lactobacillus*, *Bacteroides*, and *Clostridium* cluster IV in the colon.
